# A Study of Five Hundred Cases of Pleurisy

**Published:** 1907-08

**Authors:** 


### A STUDY OF FIVE HUNDRED CASES OF PLEURISY.

Fraley analyzes five hundred cases of pleurisy treated at the Pennsylvania Hospital from the years 1896 to 1905. He concludes his pa-

The colored race is particularly susceptible. Occupation seems to have a decided predisposing effect. Croupous pneumonia is the most frequent cause of empyema, and bronchitis, or simple exposure to dampness and cold, seems to be more frequently the exciting cause of pleurisy than tuberculasis or rheumatism (as far as clinical evidence is found.) Complications are frequent in hospital practice. Aspiration shortens appreciably the course of the disease, and usually requires to be performed but once to effect a cure. Examination of the blood is but moderately helpful and of doubtful value, as are also cytodiagnosis and inoscopy. Empyema is always dangerous, and its natural course almost invariably fatal. Resection and free drainage is the proper treatment.—American Med. Jour. May, 1907.

